# Prevalence of pulmonary tuberculosis and HIV infections and risk factors associated to tuberculosis in detained persons in Antananarivo, Madagascar

**DOI:** 10.1038/s41598-024-58309-y

**Published:** 2024-04-15

**Authors:** Fanjasoa Rakotomanana, Anou Dreyfus, Mirella M. Randrianarisoa, Mihaja Raberahona, Elodie Chevallier, Harizaka E. Andriamasy, Barivola A. Bernardson, Paulo Ranaivomanana, Francklin Ralaitsilanihasy, Miangaly Rasoamaharo, Sandro A. N. Randrianirisoa, Turibio A. Razafindranaivo, Liva Rakotobe, Andosoa Ratefiharimanana, Daniella A. Randriamanana, Harolalaina Rakotondrazanany, Bertrand Cauchoix, Laurence Baril, Niaina Rakotosamimanana, Rindra V. Randremanana

**Affiliations:** 1 Epidemiology and Clinical Research Unit, Institut Pasteur Madagascar, 101 Antananarivo, Madagascar; 2 Service des Maladies Infectieuses, Centre Hospitalier Universitaire Joseph Raseta Befelatanana, 101 Antananarivo, Madagascar; 3 Unité des Mycobactéries, Institut Pasteur Madagascar, 101 Antananarivo, Madagascar; 4 Imagerie Médicale, Hôpital Joseph Ravoahangy Andrianavalona, 101 Antananarivo, Madagascar; 5 Programme National de Lutte Contre la Tuberculose, 101 Antananarivo, Madagascar; 6 Programme National de Lutte Contre L’IST SIDA, 101 Antananarivo, Madagascar; 7 Fondation Raoul Follereau, 101 Antananarivo, Madagascar; 8 Present Address: Medical Department for Infectious Diseases, French National Agency for Medicines and Health Products Safety (ANSM), 93200 Saint Denis, France; 9 Present Address: Section of Epidemiology, Vetsuisse Faculty, University of Zurich, Winterthurestrasse 270, 8057 Zürich, Switzerland

**Keywords:** Tuberculosis, Risk factors

## Abstract

The incidence rate of tuberculosis in prisons is estimated to be 8 times greater than that in the general population in Madagascar. Our objectives were to estimate the prevalence of pulmonary tuberculosis and HIV infection among prisoners and to identify risk factors associated with tuberculosis. We conducted a cross-sectional study at the central prison of Antananarivo from March to July 2021. Individual male and female inmates aged ≥ 13 years who had lived in the prison for at least three months prior to the study period were included as participants. Acid-fast bacilli detection by microscopy and/or culture, an intradermal tuberculin test, a chest X-ray, and a rapid diagnostic orientation test for HIV were performed. Among 748 participants, 4 (0.5%) were confirmed to have pulmonary tuberculosis. Overall, 14 (1.9%) patients had “confirmed” or “probable” tuberculosis [0.90–2.84, 95% CI]. The proportion of participants with latent tuberculosis infection was 69.6% (517/743) based on a positive tuberculin test without clinical symptoms or radiography images indicating tuberculosis. Out of 745 HIV screening tests, three showed reactive results (0.4%). Age (OR = 4.4, 95% CI [1.4–14.0]) and prior tuberculosis treatment (or episodes) were found to be associated with confirmed and probable tuberculosis.

## Introduction

The global issues related to tuberculosis (TB) infection, such as the high burden in vulnerable populations, multidrug resistance and HIV coinfection have not yet been overcome. The estimated global TB incidence in 2021 was 134 (95% CI 125–143) incident cases per 100,000 people representing 10,600,000 (9,850,000–11,300,000) incident cases, whereas the total number of new and relapse cases notified was 6,431,705^1^. In the WHO African region, the incidence was estimated to be 212 incident cases per 100,000 people in 2021.

In Madagascar, the TB incidence was estimated to be 238 cases per 100,000 people in 2020 and 233 cases per 100,000 people in 2021. The effective TB treatment coverage (notified cases/estimated incidence) was 59% (41–93) in 2021 (WHO TB database); the TB case fatality (estimated mortality/estimated incidence) was 20% (10–33)^2^. Apart from the estimated TB incidence and the estimated number of new TB patients receiving treatment nationally, determining the prevalence of TB remains difficult due to the lack of available data.

Each country has specified at risk populations for TB, such as homeless persons, migrants, health care workers treating TB patients, persons living with HIV infection or other immune deficiencies, and detained persons. TB in prisons represents a common issue worldwide^3^ and evidence suggests that the TB prevalence in prisons in sub-Saharan Africa is underestimated^4^. Given the confined space shared by many detained persons, the TB contact rate and transmission risk may be very high, leading to a high prevalence of TB in prisons. Compared to that in general population, the prevalence of TB in prisons is also underdocumented in Madagascar.

Compared to that in other African countries, the prevalence of HIV in Madagascar is estimated to be 0.4% which is rather low^5^; however, the risk of HIV among incarcerated people may be greater than in the general population^6^. The National Tuberculosis Control Program recommends systematic screening for HIV infection along with TB diagnosis to understand the risk of comorbid. In general, the risk of HIV transmission in prisons has been reported to be very high^7^. In Madagascar, however, the prevalence is poorly understood, as few studies have reported data on this issue among incarcerated people. TB and HIV infection, also called the “cursed couple”, are both associated with stigma, and a lack of diagnosis and medical care.

The aim of this study was to describe the prevalence of TB (active disease and latent infection) and HIV infection among incarcerated people and the associated risk factors. The results could contribute to the knowledge of the prevalence of TB in prisons and to the development of possible preventive measures.

## Methods

### Study population and study site description

Madagascar has a population of approximately 26 million people and a total of 44 prisons. The present study was conducted in the Antanimora prison in Antananarivo, the capital of Madagascar, which is also known as “Maison Central d’Antanimora” (MCA). This prison has overcrowding and undernourishment problems^8^. It was difficult to accurately determine the number of detainees during the study period (March–July 2021). The detained population fluctuated considerably (new arrivals, releases, transfers, etc.), with a high rate of pretrial detention. According to data collected by the Malagasy Ministry of Justice, approximately 37% of detainees were sentenced; approximately 63% had been charged and were still awaiting trial. In 2021, the average prison population was 3840 people, of whom 89% were male. Approximately 3% of the male detainees were minors, and approximately 2.5% were female. The detainees were distributed in five main areas called “quarters”: one for men, one for women and female minors, one for male minors and two dedicated quarters (one for men and one for women). These dedicated quarters accommodate for detained persons who can afford for better living conditions.

### Study design, sample size and sampling frame

From March to July 2021, a cross-sectional study was conducted in the MCA to determine the prevalence of TB (active disease and latent infection) and HIV and to assess the risk factors associated with these diseases. To estimate a prevalence of 10% (such as for TB disease) with an alpha precision of 0.05 and a confidence interval of 95%, a sample size of 139 was needed. However, if the estimated prevalence was 60% (latent TB), a sample size of 369 was required^9^. To estimate risk factors for TB with an estimated 10% TB prevalence, a power of 90%, a 1/3 ratio of exposed to unexposed and a minimum relative risk of 2 to be attained, we needed 724 participants^10^. A sample size of 750 detainees was selected to ensure representativeness and statistical power^10^. For HIV, a sample size of 16 was required to estimate a prevalence of 1% with an alpha of 0.05 and a confidence interval of 95%.

Since the number of inmates in the different quarters varied widely, especially between the male and female quarters, a proportional random stratification of the participants per quarter from a list of detainees was performed to obtain representative samples of the inmate population. The inclusion criteria were as follows: individual male or female inmates with ≥ 13 years of age, which is the legal age for incarceration; and presence in prison for at least three months prior to the study period.

Participation was completely voluntary; individuals who were ineligible and those who refused to participate were replaced to obtain the number of the expected sample size. The participants were first informed about the study verbally by their supervisors (who were also inmates) about the study and then by the research team (verbally and in writing). The study began after participants signed the informed consent forms. Minors were personally asked if they were willing to participate. If a minor personally consented, their consent form was signed by a special educator who legally represented their parent or legal guardian.

### Data collection

The study participants underwent clinical and paraclinical examinations. The Redcap electronic data capture tool (https://projectredcap.org/software) was used to collect the data.

Tuberculin skin test (TST; Mantoux test) was performed by intradermal injection of 0.1 ml of the test dose. The diameter of the skin induration was measured after 48–72 h. A diameter of less than 10 mm was considered negative in a country with a high prevalence of TB, such as Madagascar, where the “Bacille de Calmette et Guerin” (BCG) vaccine is part of the mandatory vaccination program during the first year of childhood. An induration ≥ 10 mm was considered significantly positive (≥ 5 mm for the HIV-positive individuals)^11^.

Chest radiography was performed using a MinXray® machine (except if contraindicated or for pregnant women). The results were first classified into normal and abnormal chest images. The abnormal images were described according to the type of lesions and the suspected diagnosis. In the case of atypical images, a second interpretation was suggested. Information about clinical symptoms, biological results or contact with other TB patients were taken into account for image interpretation.

A bacteriological diagnosis of acid-fast bacilli (culture and microscopy) was performed for participants who had respiratory symptoms and for whom two samples of sputum could be obtained by coughing.

In the Mycobacteria Unit, smear microscopy positive for acid-fast bacteria and Lowenstein–Jensen (LJ) solid medium or Mycobacteria Growth Indicator Tube liquid medium culture for sputum was performed. Phenotypic drug-susceptibility testing for first-line antituberculosis drugs (isoniazid, rifampicin, ethambutol, pyrazinamide, and streptomycin) was performed by the antibiogram proportion method using LJ solid medium based on WHO recommendations^12^.

All confirmed TB cases were referred to the local Diagnosis and Treatment Center for TB treatment according to the National Tuberculosis Control Program therapeutic schemes.

An HIV test was systematically performed on the basis of voluntary participation. An immunochromatographic test for the qualitative detection of antibodies against HIV-1 and HIV-2 called (Alere Determine™ HIV-1/2) was used to screen for HIV cases. If the first HIV test result was positive, a doctor from the HIV referral health center came to the prison health center to confirm the test before treatment according to the recommendation from the National HIV Program.

### Case definitions of pulmonary tuberculosis

The case definitions of pulmonary TB were based on bacteriological test results, clinical signs and symptoms associated with TB, and radiography and/or skin test results.

Patients with confirmed active TB (disease) presented with a productive cough and had positive microscopy results and/or sputum cultures for *Mycobacterium tuberculosis*.

Patients with a probable active TB had an episode of coughing (> 14 days) associated with one or more symptoms compatible with TB (fever, night sweats, weight loss, loss of appetite, chest pain, dyspnea) and an abnormal chest X-ray indicating progressive TB.

Participants with suspected TB case were defined as persons with an episode of cough (> 14 days) associated with one or more symptoms compatible with TB (fever, night sweats, weight loss, loss of appetite, chest pain, dyspnea) but without an abnormal chest radiograph indicating progressive TB, or as persons with only an abnormal chest radiograph compatible with TB but without clinical signs or symptoms.

Persons who had a positive skin test (induration diameter ≥ 10 mm; ≥ 5 mm for an HIV-positive person) and who did not belong to the categories defined above were classified as latent TB infection participants. Latent TB infection (LTBI) is characterized by TB infection without clinical symptoms or radiographic images associated with TB diagnosis^13^.

### Statistical analysis

The number and percentage of participating detainees with diagnostic test results (TST, chest radiography, direct microscopy and culture, HIV tests) were estimated.

The total study population and the participants with the outcome “confirmed or probable TB” were stratified by categories of potential risk factors (“exposure variables”), including demographics (i.e., age, sex, education level), time in prison, and contact with other TB patients in detention centers. Fisher’s exact test was performed if there were fewer than five data points in a cell of the 2 × 2 table; otherwise, the chi-square test was used to determine whether there was a statistically significant difference (*p* ≤ 0.05) between the categories of exposure variables.

We applied univariate logistic regression analysis to assess the association between the outcomes of “confirmed or probable TB” and the potential risk factors (explanatory variables). We then performed a multivariate logistic regression analysis using a manual backward selection approach. Exposure variables were entered into the multivariable model if the *p* value was < 0.2 in the univariate analysis or if the variable was considered a potential confounding factor. Correlations between exposure variables were tested by checking their associations. If the chi-square/Fisher exact test was statistically significant (*p* ≤ 0.05), only one of the associated exposure variables was entered in the multivariable model at one time. Exposure variables were kept in the multivariable model if the likelihood ratio test was statistically significant (*p* ≤ 0.05) or if the Akaike information criterion (AIC) was lower than that of the nested model. Confounding factors were retained in the model if their presence changed the OR of another exposure variable in the final model by ≥ 20%.

Since detainees lived together in a specified quarter, separated from detainees from other quarters, we constructed a multivariable mixed model of the binomial family, controlling for clustering on the quartier level (block = random effect).

To assess whether the data fit the model, post modeling model diagnostics were conducted using the Hosmer‒Lemeshow test and Pearson residual and residual deviance tests were conducted.

Given the voluntary nature of the study, we compared the included study participants and those who would have been eligible but refused/were unable to participate based on the exposure variables “age” and “sex”. A difference would indicate that the two populations differed and that a potential selection bias may have been introduced.

### Ethical approval

This cross-sectional study was performed in accordance with the principles of the Declaration of Helsinki and was approved by the Ministry of Public Health via the National Ethical Committee of Biomedical Research (No. 042 MSANP/CERBM) on June 13th, 2019.

## Results

### Study population

**Figure 1:**
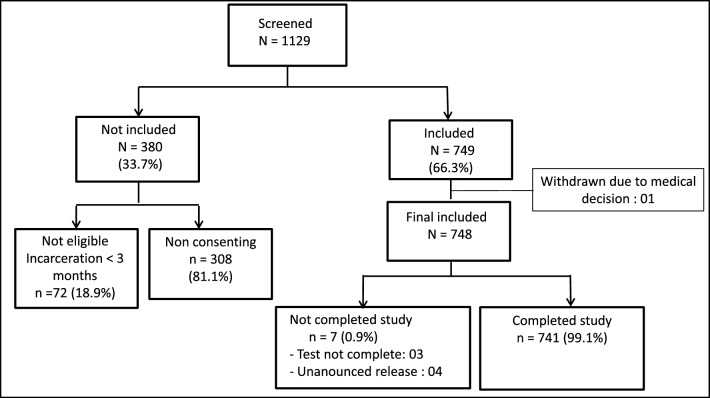
Flowchart of inclusion, exclusion and final number of study participants.

During the study, 1129 detainees were screened based on the random sampling list. A total of 749 out of approximately 3840 incarcerated persons (on average), were eligible and consented to participate in the study. The principal reason for refusal to participate was the perception of being healthy and therefore not needing to be examined. Other participants did not trust the researchers. One participant stopped participating due to a medical decision, and 748 participants completed the clinical examinations. Figure 1 illustrates the data collection flowchart.

The sex ratio (male/female) of the participants was 8.8. The mean age of the adults was 32 years, with a range from 18 to 73 years. Of the total participants, 2% (15/748) were minors. Up to 92.5% (692/748) of the participants were experiencing their first incarceration. The median duration of imprisonment was 1.1 years. Table 1 shows the socio-demographic characteristics of the participants.

**Table 1:** Socio-demographic characteristics of the participants, March–July 2021, MCA, Madagascar.

	Total (%) N = 748
Sex	
Female	75 (10.0%)
Male	673 (90.0%)
Age group	
Age ≥ 18 years	733 (98.0%)
Age < 18 years	15 (2.0%)
Married	
No	559 (74.7%)
Yes	130 (17.4%)
Education level	
University	62 (8.3%)
High school	352 (47.1%)
Elementary	296 (39.6%)
Illiterate	38 (5.1%)
First incarceration	
No	30 (4.0%)
Yes	718 (96%)
Unemployed	
No	711 (95.1%)
Yes	37 (4.9%)

### Diagnostic tests results

The prison study setting was challenging, as some prisoners agreed to participate in one test but refused to participate in the others. For example, some participants underwent chest X-ray, but refused the injection (TST). Others agreed to undergo the TSTs but were absent for the measurement of the induration. There were variations in TST reading because the prison is not accessible during the week-end. The TST administration was scheduled considering this issue. Some test ‘s readings were gathered to avoid week-end. A few study participants were suddenly released or they missed a test because of their trial.

A total of 747/748 TSTs were performed, of which 743 (99.9%) were read. Pulmonary X-rays were available for 741 participants. A total of 48 participants reported having respiratory symptoms; of these participants, 35 had sputum samples collected for acid-fast bacilli detection. Among 748 participants, 745 underwent HIV tests (99.5%). Overall, 741/748 participants (99%) had completed paraclinical data.

Even if a test result was missing for a participant, he or she was not excluded from the study; their data were still used in the analysis when available.

### Prevalence of tuberculosis and HIV

We included 748 inmates in our study, in which 741 participants completed all the clinical and paraclinical examinations. Among the 748 participants, four (0.5%) [0.01–1.06, 95% CI] were confirmed to have pulmonary TB and had positive microscopy and/or culture results. Ten of the 748 participants (1.3%) [0.51–2.16, 95% CI] were classified as having “probable TB”, because they presented with cough (≥ 14 days), had one or more symptoms compatible with TB and had a chest radiograph indicating progressive TB. The overall number of confirmed and “probable” TB patients was 14/748 (1.9%) [0.90–2.84, 95% CI]. The proportion of latent TB infection cases was 69.6% (517/743) [66.27–72.89, 95% CI] based on a positive tuberculin test without clinical symptoms or radiographic evidence of TB.

Three out of 745 (0.4%) participants had a positive HIV test (95% CI [0.1–1.2]). No TB/HIV coinfection was observed. Table 2 shows the prevalence of TB and the prevalence of HIV.

**Table 2:** Prevalence of TB and prevalence of HIV by sex and by age, March–July 2021, MCA, Madagascar.

	Total N = 748 (100%)	Tuberculosis		HIV test result			
		No N = 734 (98.1%)	Yes N = 14 (1.9%)	Total N = 748 (100%)	Non-reactive N = 742 (99.2%)	Reactive N = 3 (0.4%)	Not done N = 3 (0.4%)
Sex							
Female	75 (10.0%)	75 (10.0%)	0 (0.0%)	75 (10.0%)	75 (10.0%)	0 (0.0%)	0 (0.0%)
Male	673 (90.0%)	659 (88.1%)	14 (1.9%)	673 (90.0%)	667 (89.2%)	3 (0.4%)	3 (0.4%)
Age							
≥ 18 years	733 (98.0%)	719 (96.1%)	14 (1.9%)	733 (98.0%)	727 (97.2%)	3 (0.4%)	3 (0.4%)
< 18 years	15 (2.0%)	15 (2.0%)	0 (0.0%)	15 (2.0%)	15 (2.0%)	0 (0.0%)	0 (0.0%)

### Risk factors for pulmonary tuberculosis

In the univariable analysis, in which 741 participants were included (the others had missing data) the exposure variables “age” (OR = 5.6, 95% CI [1.9–18.5]), “history of a previous stay in another prison” (OR = 7.1, 95% CI [1.6–24.5), “prior history of tuberculosis” (OR = 8.4, 95% CI [1.8–29.1]), and “history of TB treatment” (OR = 9.7, 95% CI [2.5–31.0]) were statistically significantly associated with the outcome “being a confirmed or probable TB patients” (Tables S1 and S2).

In addition to the abovementioned variables, the following variables with a p value < 0.2 in the univariable analysis were assessed in the multivariable logistic regression model: “sex”, “age category”, “number of TB patients living in the same room”, “transient admission to another correctional facility”, and “marital status”. Furthermore, we added either the variable “history of treatment with anti-TB drugs” (AIC: 128.93) or “history of a TB diagnosis” (AIC 132.30) to the model. These two variables were strongly associated, and only one could be tested at a time. The final multivariate model with the best fit included the variables “age category” and “history of treatment with anti-TB drugs” (Table 3). Detained persons 40 years of age or older were 4.4 times more likely to have confirmed or probable TB than were detained persons younger than 40 years of age, controlling for the effect of “having ever received TB treatment at any time in their life”. Detainees who “had ever received TB treatment at any time in their life” were 6.3 times more likely to have confirmed or probable TB than detained persons who had never been treated, before controlling for the effect of age. Both ORs had a rather large confidence interval, with a possible outcome of almost no effect (1.5 and 1.6) to a high effect (15.1 and 21.3).

**Table 3:** Risk factors associated with the outcome “being a confirmed or probable TB patient” in the final multivariable logistic regression model in persons (N = 747**) detained from the Antanimora prison in Antananarivo, Madagascar; cross-sectional study from March–July 2021.

Exposure variable categories	Confirmed or probable TB case		Crude odds ratio (95% CI)*	Adjusted odds ratio (95% CI)*	p-value
	Yes N = 14	No N = 733			
Age group					
Age 13–39 years	5 (35.7%)	556 (75.9%)	Ref	Ref	0.01
Age ≥ 40 years	9 (64.3%)	177 (24.1%)	5.6 (1.9–18.6)	4.4 (1.4–14.0)	
Ever received TB treatment at any time in your life					
No	10 (71.4%)	704 (96.0%)	Ref	Ref	0.004
Yes	4 (28.6%)	29 (4.0%)	9.7 (2.5–31.0)	6.3 (1.8–22.5)	

*95% CI 95% confidence interval; variables included in the stepwise multiple logistic regression were sex, age category, number of TB patients living in the same room, history of treatment with antituberculosis drugs, OR history of TB diagnosis (the two variables were significantly associated, hence only one of two variables were tested at a time), transient admission to another correctional facility, and marital status.

**One observation was dropped by the model. AIC = 130.8

The multivariable mixed model that controlled for clustering at the prison block level did not show a difference in relative risk; therefore, we kept the simpler multivariable logistic regression model, which did not control for the effect of clustering.

The p-values of the Hosmer‒Lemeshow, Pearson residual, and residual deviance tests were 0.999, 0.609 and 1, respectively, indicating a sufficient model fit of the data.

### Selection bias

The sex distribution significantly differed between the included and nonincluded study populations (p-value < 0.001). Among the included study participants, 10% (75/748) were female and 90% were male (673/748); among the nonincluded individuals, only 1.6% (5/308) were female and 98.4% were male. There was also a slight age difference (median of 29.5 vs. 31 years) between the included and nonincluded individuals.

## Discussion

The present study was designed to determine the prevalence of different stages of TB and of HIV in detention centers and the associated risk factors in Madagascar.

In this TB prevalence study in the Madagascar’s main prison, four individuals with confirmed TB were identified among the 748 study participants (0.53%), extrapolated to a prevalence of 534/100,000 persons. We are unable to compare our TB prevalence data with the current TB prevalence in the general population, as unfortunately, no published data for the same period are available. A study conducted in the central detention center of Antanimora in 1993 showed an estimated prevalence of 2.4%, while the maximum estimated prevalence for the country was 0.3% (i.e., 8 times lower)^14^. A study conducted in this prison in 1994–1995 estimated that the TB prevalence was 16 times greater in detainees than in the general population^15^. However, in 2014, a report on a national nutrition protocol for TB patients and/or people living with HIV issued by the Malagasy Ministry of Health and the World Food Program (WFP) estimated a TB prevalence of 413 cases per 100,000 people in 2013^16^ (the survey that was used to collect these data is unfortunately not described). By comparing the TB prevalence of our study (534/100,000) to the TB prevalence of 413 cases/100,000 people estimated in 2013, we could conclude that the TB prevalence found in our study was not much greater than that of the general population. However, given that the data were collected nine years before and the survey design is not described, this conclusion should be interpreted with caution.

TB prevalence studies have been conducted in some regions of Africa. A study conducted in a detention center in northwest Ethiopia in 2011 revealed a very high TB prevalence, with 1482.3 cases per 100,000 smear-positive individuals with suspected TB^17^; another study conducted in southern Ethiopia in 2013 estimated 349.2 TB cases per 100,000 people^18^. O’Grady et al. (2011) reported that the TB prevalence in prisons can be up to 50 times greater than the national average^7^. A study conducted in Niger prisons for three years (2008–2010) showed a 0.8% prevalence of pulmonary TB^19^. These studies confirmed that TB is prevalent in prisons and that the prevalence of the TB in the detainee population may be greater than that in the general population. However, it is not possible to separate some new cases from in situ transmission or activation of LTBI activation prior to incarceration.

There are no TB prevalence studies in the general population in Madagascar. The cost of conducting a national prevalence survey and the current limited accessibility of 30% of the country’s regions reduce the representativeness of the national prevalence survey^20^.

In countries where TB is endemic and the TB incidence is high, the prevalence of LTBI is greater than in countries with a low TB incidence. We found an LTBI prevalence of 69.6% in the study population. The high prevalence of LTBI confirms the high burden of TB in Madagascar. We could not find published data on the prevalence of latent TB in Madagascar. Approximately 5–10% of people with LTBI are at risk of developing active TB^21^. Inmates with suspected and probable TB require more attention and follow-up regarding the risk of developing or transmitting the disease to other inmates. In addition, the prisons are not completely closed: many detained persons are not condemned and are released after a brief delay. Rasolofo-Razanamparany et al.^15^ showed that some of the TB strain clusters from the Antanimora prison were also found in nonincarcerated persons in the city of Antananarivo. People typically become infected when they are young, and most detained persons will not be in detention centers for more than a few years. Our study revealed that the median duration of incarceration was 1.1 years. Therefore, detainees are part of the population, and the diseases that are found in prisons are the same as those found outside of prisons.

While the movements of detained persons are strictly controlled in prisons, contact with the outside world is still possible due to staff movement and visits by relatives, family members, and NGO workers who are regularly in contact with the detained persons. Hence, detainees may be exposed while in prison and may infect others or become infected. Furthermore, they may expose the general population to TB once they are released^22^. Therefore, it is crucial to detect cases of TB in detained persons and treat them for TB while they are still in prison.

Two variables, “age category” and “history of treatment with anti-TB drugs”, were strongly associated with “confirmed or probable” TB cases in the multivariable model and hence identified as potential risk factors for confirmed and probable TB.

Age is generally associated with TB because the risk of infection and contact with infected persons increases with time^23^. Moreover, the immune system becomes weaker with age, and latent TB may progress to disease^24^. Why was the exposure variable “having a history of TB treatment” a risk factor in the multivariable analysis? One reason could be that people who were previously ill had a predisposition for infection in an environment of high infection pressure (i.e., a weak immune system); therefore, they experienced relapse. Another reason could be that they had never been cured of TB (i.e., their treatment may have been interrupted). In any case, the results of the multivariable analysis should be interpreted with caution, given the low case numbers and the multitude of risk factors. The uncertainty is also reflected in a rather large confidence intervals of the odds ratios.

The WHO case definitions for TB included “confirmed TB” and “latent TB infection”^13^. In our study, however, we further defined “suspected” and “probable” cases. The main reason for this was the limited clinical and diagnostic resources in the prisons; therefore, some participants did not receive all tests and could not be clearly classified into these two categories. For example, sputum samples could only be collected from detainees who could provide a sputum sample spontaneously by coughing. Therefore, some patients only had a “positive” X-ray diagnosis without microbiological confirmation. They would not have been considered when using the classical case definition, even though their odds of having TB were increased. Certain symptoms commonly associated with TB, such as emaciation, fever, and night sweats, could not be considered specific because they may be provoked by the living conditions in prisons. Chest radiography may constitute an alternative to the lack of diagnostic tools related to the restriction of displacement outside the prisons, but the high cost of mobile equipment limits its use in prisons. For the same reason, the prevalence of confirmed TB cases in our study is most likely an underestimate, as only 35 people were able to provide a sputum sample. Consequently, we decided to include the probable cases in the outcome variable in the analysis of risk factors.

Another limitation is the use of smear microscopy instead of the GenXpert method. This limitation likely impacts the accuracy of the TB prevalence estimates provided by the study. However, the GenXpert is being used in another follow-up study.

Very few women participated in the study, but this reflects the demographics of the prison population. Therefore, the numbers we found cannot be extrapolated to women. This phenomenon may represent a selection bias that is based on the natural distribution of male and female prisoners.

## Conclusion

We observed a significantly high prevalence of TB within the Antanimora prison in Antananarivo, with an active (confirmed) TB disease prevalence of 0.5% and an overall “probable” and “confirmed” TB prevalence of 1.9%. The results showed a high risk of TB in prison, particularly for people over 40. This means that TB screening and surveillance needs to be increased from this age onwards. The Malagasy Ministry of Public Health and the Ministry of Justice must collaborate to enhance medical services within prisons, particularly for TB care. The two ministries need to strengthen a referral strategy for patients. This will prevent patients from being lost once they are released from prison.

Due to the absence of prevalence data in the general population, we cannot definitively establish a greater infection risk within prisons compared to population outside prisons. However, considering our findings and the prison living conditions, we anticipate a heightened risk of TB transmission among detainees in prison, as well as an increased risk of transmission between prisoners and the general population (and vice versa) during and after detention, as prisoners are not completely isolated from the external community.

The external population may pose a risk to vulnerable prisoners, whose immune systems may be compromised due to nutritional deficiencies and a lack of medical care. Hence, it is imperative for prisoners to undergo regular TB testing and receive medical care while incarcerated. This measure will not only enhance the quality of life and healthcare for inmates but also safeguard the general population.

### Supplementary Information

Supplementary Tables.

## Data Availability

The datasets generated or analyzed during the current study are available from the corresponding author on reasonable request.
