# Supplementary material for: Prevalence of pulmonary tuberculosis and HIV infections and risk factors associated to tuberculosis in detained persons in Antananarivo, Madagascar
Source: Sci Rep. 2024 Apr 15;14:8640. doi: 10.1038/s41598-024-58309-y (PMC11018834; doi:10.1038/s41598-024-58309-y)
Supplement: Supplementary file 1 — Supplementary Tables. [file 41598_2024_58309_MOESM1_ESM.pdf]

Supplementary tables

Table S1: Description of the study population according to socio-demographic characteristics, detention centers quartier, body mass index (BMI), rapid HIV test result, and incarceration history/information (= exposure variables). Association between these exposure variables and being a confirmed or probable TB case (odds ratio); cross-sectional study in the detention center of Antanimora, Madagascar, March - July 2021

**Exposure variable** Categories	**Total study population (N=748)**		**Confirmed or probable TB case (N=14)**				
	**n (%)/median (IQR)**	**p-value**	**n (%)/median (IQR)**	**p-value[3]**	**Odds Ratio**	**95% CI****	**p-value[3]**
Sex		< 0.01		NA[4]			NA[4]
Female	75 (10.1%)		0 (0.0%)		NA[4]	NA[4]	
Male	673 (90.0%)		14 (100.0%)		NA[4]	NA[4]	
Age group		< 0.01		**0.002**			0.002
Age 13 to 39 years	561 (75.0%)		5 (35.7%)		Ref		
Age ≥ 40 years	187 (25.0%)		9 (64.3%)		5.6	1.9-18.5	
Detention centers quarter		< 0.01		NA[4]	NA[4]	NA[4]	NA[4]
Adult	721 (96.4%)		14 (100.0%)		NA[4]	NA[4]	
Minor	21 (2.8%)		0 (0.0%)		NA[4]	NA[4]	
Annex	6 (0.8%)		0 (0.0%)		NA[4]	NA[4]	
Education level		< 0.01		0.394			0.629
University	62 (8.3%)		1 (7.1%)		Ref		
High school	352 (47.1%)		6 (42.9%)		1.1	0.2-20.2	
Elementary	296 (39.6%)		5 (35.7%)		1.0	0.2-20.3	
Illiterate	38 (5.1%)		2 (14.3%)		3.4	0.3-74.4	

Exposure variable Categories	Total study population (N=748)		Confirmed or probable TB case (N=14)				
	**n (%)/median (IQR)**	**p-value**	**n (%)/median (IQR)**	**p-value[3]**	**Odds Ratio**	**95% CI****	**p-value[3]**
Body mass index (BMI)[1]	20.5 [19.1 - 22.4]	-	19.3 [17.6 - 20.2]	0.685	1.0	1.0-1.0	0.692
BMI[1,2]				0.5167			0.671
18,5-25	559 (74.7%)	< 0.01	9 (64.3%)		Ref		
< 18.5	130 (17.4%)		4 (28.6%)		1.9	0.5- 6.1	
25-30	47 (6.3%)		1 (7.1%)		1.3	0.1-7.3	
≥30	12 (1.6%)		0 (0.0%)		NA[4]	NA[4]	
Rapid HIV test result[2]		< 0.01		NA[4]			NA[4]
Non-reactive	742 (99.6%)		13 (100.0%)		NA[4]	NA[4]	
Reactive	3 (0.4%)		0 (0.0%)		NA[4]	NA[4]	
Not done	3		1				
First incarceration		< 0.01		NA[4]			NA[4]
No	57 (7.6%)		0 (0.0%)		NA[4]	NA[4]	
Yes	691 (92.4%)		14 (100.0%)		NA[4]	NA[4]	
Length of time since incarceration (year)	1.1 [0.5 - 2.1]		1.3 [0.7 - 2.3]	0.419	1.1	0.9- 1.2	0.238
History of having stayed in another detention center		< 0.01		**0.016**			0.015

Exposure variable<br>Categories	Total study population (N=748)		Confirmed or probable TB case (N=14)				
	**n (%)/median (IQR)**	**p-value**	**n (%)/median (IQR)**	**p-value[3]**	**Odds Ratio**	**95% CI****	**p-value[3]**
No	718 (96.0%)		11 (78.6%)		Ref	6	
Yes	30 (4.0%)		3 (21.4%)		7.1	1.6-24.5	
Duration of admission in another penitentiary (year)*	2.5 [1.0 - 5.0]		9.0 [5.0 - 18.0]	**0.176**	1.2	1.0-1.4	0.048
Number of inmates living in the same cell	127 [88.0 - 170.0]		130 [82 - 177]	0.661	1.0	1.0- 1.0	0.776
A person with tuberculosis in the same "quarter"		< 0.01		NA[4]			NA[4]
No	27 (3.6%)		0 (0.0%)		NA[4]	NA[4]	
Yes	721 (96.4%)		14 (100.0%)		NA[4]	NA[4]	
At least one TB patient in the same detention center cell		0.534		0.373			0.308
No	382 (51.1%)		5 (35.7%)		Ref		
Yes	366 (48.9%)		9 (64.3%)		1.90	0.65- 6.23	
Number of TB patients in	0.0 [1.0 - 2.0]		1.5 [0.0 - 3.0]	**0.189**	1.3	0.9- 1.9	0.243

Exposure variable Categories	Total study population (N=748)		Confirmed or probable TB case (N=14)				
	n (%)/median (IQR)	p-value	n (%)/median (IQR)	p-value[3]	Odds Ratio	95% CI**	p-value[3]
the same room							

[1] *Fisher exact or chi-square test or Wilcox test;* [2] *1 observation missing;* [3] *simple logistic regression;* [4] *the model could not run because of missing observations*

** Due to the low number of observations, the variable was not added into the multiple logistic regression*

*** 95% CI: 95% confidence interval*

Table S2: Description of the study population according to substance use, medical history, and clinical signs and symptoms (= exposure variables). Association between these exposure variables and being a confirmed or probable TB case (odds ratio); cross-sectional study in the detention center of Antanimora, Madagascar, March - July 2021

Exposure variable Categories	Total study population (N=748)		Confirmed or probable TB case (N=14)				
	n (%)/median (IQR)	p-value	n (%)/median (IQR)	p-value[9]	Odds Ratio	95% CI**	p-value[10]
Consumption of psychoactive substances[1]		< 0.01		1.000			0.471
No	650 (86.9%)		13 (92.9%)		Ref		
Yes	98 (13.1%)		1 (7.1%)		0.5	0.0-2.6	
Cigarettes[1]		< 0.01		0.392			0.488
No	326 (43.6%)		5 (35.7%)		Ref		
Yes	342 (45.7%)		6 (42.9%)		1.1	0.3-4.0	

Exposure variable<br>Categories	Total study population (N=748)		Confirmed or probable TB case (N=14)				
	n (%)/median (IQR)	p-value	n (%)/median (IQR)	p-value[9]	Odds Ratio	95% CI**	p-value[10]
Quit	80 (10.7%)		3 (21.4%)		2.5	0.5-10.4	
Chew tobacco [2,3]		< 0.01		1.000			0.749
No	241 (73.9%)		4 (80.0%)		Ref		
Yes	85 (26.1%)		1 (20.0%)		0.7	0.0-4.9	
Alcohol[1]		< 0.01		0.226			0.309
No	659 (88.1%)		11 (78.6%)		Ref		
Yes	89 (11.9%)		3 (21.4%)		2.1	0.5-6.7	
Prior history of tuberculosis [4,5]		< 0.01		**0.011**			0.01
No	718 (96.5%)		11 (78.6%)		Ref		
Yes	26 (3.5%)		3 (21.4%)		8.4	1.8-29.1	
Duration of previous TB disease (months)[6]	6.0 [5.5-8.5]	-	8.0 (4.5 – 13.0)	0.626	1.1	0.8-1.5	0.416
Ever received TB treatment at any time in your life [7,8]		< 0.01		**0.002**			0.002
No	714 (95.6%)		10 (71.4%)		Ref		
Yes	33 (4.4%) *		4 (28.6%)		9.7	2.5-31.0	
Previously received		< 0.01		NA[11]			NA[11]

Exposure variable Categories	Total study population (N=748)		Confirmed or probable TB case (N=14)				
	n (%)/median (IQR)	p-value	n (%)/median (IQR)	p-value[9]	Odds Ratio	95% CI**	p-value[10]
antiretroviral therapy [1]							
No	747 (99.9%)		14 (100.0%)		NA[11]	NA[11]	
Yes	1 (0.1%)		0 (0.0%)		NA[11]	NA[11]	
Using prednisolone or other corticosteroid [1]		< 0.01		**0.173**			0.174
No	738 (98.7%)		13 (92.9%)		Ref		
Yes	10 (1.3%)		1 (7.1%)		6.2	0.3-36.8	
Fever [1]		< 0.01		< 0.001	NA[12]	NA[12]	NA[12]
No	745 (99.6%)		12 (85.7%)				
Yes	3 (0.4%)		2 (14.3%)				
Expectoration[1]		< 0.01		< 0.001	NA[12]	NA[12]	NA[12]
No	653 (87.3%)		4 (28.6%)				
Yes	95 (12.7%)		10 (71.4%)				
Weight loss [1]		< 0.01		0.002	NA[12]	NA[12]	NA[12]
No	559 (74.7%)		5 (35.7%)				
Yes	189 (25.3%)		9 (64.3%)				
Night sweats [1]		< 0.01		< 0.001	NA[12]	NA[12]	NA[12]
No	727 (97.2%)		10 (71.4%)				

Exposure variable<br>Categories	Total study population (N=748)		Confirmed or probable TB case (N=14)				
	**n (%)/median (IQR)**	**p-value**	**n (%)/median (IQR)**	**p-value[9]**	**Odds Ratio**	**95% CI****	**p-value[10]**
Yes	21 (2.8%)		4 (28.6%)				
Loss of appetite[1]		< 0.01		0.141	NA[12]	NA[12]	NA[12]
No	740 (98.9%)		13 (92.9%)				
Yes	8 (1.1%)		1 (7.1%)				
Respiratory signs[1]		< 0.01		< 0.001	NA[12]	NA[12]	NA[12]
No	700 (93.6%)		7 (50.0%)				
Yes	48 (6.4%)		7 (50.0%)				
Chest pain[1]		< 0.01		< 0.001	NA[12]	NA[12]	NA[12]
No	714 (95.5%)		8 (57.1%)				
Yes	34 (4.5%)		6 (42.9%)				
Shortness of breath[1]		< 0.01		< 0.001	NA[12]	NA[12]	NA[12]
No	726 (97.1%)		10 (71.4%)				
Yes	22 (2.9%)		4 (28.6%)				

[1] One observation missing; [2] 422 observations missing for the total study population; [3] 10 observations missing for confirmed or probable TB case; [4] 2 observations missing for the total study population; [5] 1 observation missing for the confirmed or probable TB case;
[6] History of tuberculosis: 726 missing observations for the total study population and 11 missing observations for being a confirmed or probable TB case; [7] 2 observations missing for the total study population;
[8] 1 observation missing for being a confirmed or probable TB case;
[9] Fisher's exact or chi-square test or Wilcox test; [10] Simple logistic regression; [11] NA not applicable because of 0 data points in one category;

[12] NA not applicable, as clinical signs and symptoms are part of the case definition for being a probable pulmonary TB case

** While 29 study participants claimed to have had TB, 33 persons, hence four more, claimed that they had received TB treatment before. Unfortunately, the reason for this incongruence is unknown. Perhaps those who received treatment received it for diagnostic purposes.*

*** 95% CI: 95% confidence interval*
